# Real‐time motion detection of functional MRI data

**DOI:** 10.1120/jacmp.v5i2.1969

**Published:** 2004-08-16

**Authors:** Theodore R. Steger, Edward F. Jackson

**Affiliations:** ^1^ Department of Imaging Physics The University of Texas M. D. Anderson, Cancer Center Unit 56, 1515 Holcombe Blvd. Houston Texas 77030

**Keywords:** functional magnetic resonance imaging, neurosurgical planning, motion detection

## Abstract

The objective of this work was to implement a motion‐detection algorithm on a commercial real‐time functional magnetic resonance imaging (fMRI) processing package for neurosurgical planning applications. A real‐time motion detection module was implemented on a commercial real‐time processing package. Simulated functional data sets with introduced translational, in‐plane rotational, and through‐plane rotational motion were created. The coefficient of variation (COV) of the center of intensity was used as a motion quantification metric. Coefficients of variation were calculated before and after image registration to determine the effectiveness of the motion correction; the limits of correctability were also determined. The motion‐detection module allowed for real‐time quantification of the motion in an fMRI experiment. Along with knowledge of the limits of correctability, this enables determination of whether an experiment needs to be reacquired while the patient is in the scanner. This study establishes the feasibility of using real‐time motion detection for presurgical planning fMRI and establishes the limits of correctable motion.

PACS number: 87.61.‐c

## I. INTRODUCTION

Functional magnetic resonance imaging (fMRI), a noninvasive technique for performing functional brain mapping, is playing an increasingly important role in presurgical planning.^(1)^ Prior to surgery, the patient is asked to perform a battery of functional tasks in order to determine the location of relevant functional centers relative to the tumor or lesion to be resected. The functional information is used preoperatively for risk management and planning the surgical trajectory and can be used intraoperatively to guide tumor resection.^(1)^ Among the difficulties encountered when performing clinical fMRI are the increased likelihood of subject motion, the decreased likelihood of subject compliance, and the requirement that the results be generated rapidly to allow the data to be available to the surgeon.

In traditional fMRI, the data are acquired and transferred to a separate workstation where offline statistical analysis, motion correction, and additional processing are performed. This offline processing is generally done after the examination is complete, meaning that the reacquisition of data corrupted by excessive motion or lack of patient compliance requires recall of the patient. In a busy clinical setting, fMRI data are often acquired the afternoon prior to an early morning surgery, making patient recall impractical or impossible.

Real‐time processing of fMRI data has recently been introduced in a number of commercially available software packages. Real‐time processing is useful in that it allows the map of functional activation to be displayed immediately following the fMRI experiment, while the patient is still in the scanner. If the data are deemed to be inadequate, new data may be acquired immediately.

In a review of their clinical fMRI experience, Krings et al. reported that presurgical fMRI failure was most frequently caused by head motion artifacts.^(1)^ Real‐time fMRI provides a purely qualitative assessment of motion based on the presence of areas of spurious activation around the periphery of the brain that is unique to an image corrupted by stimulus‐correlated motion.^(2)^

Although this artifact can be identified immediately via real‐time processing, a difficulty arises in the fact that motion beyond a certain magnitude cannot be sufficiently corrected. For example, Krings et al. concluded that for their in‐house image‐registration package, motion greater than 2 mm could not be adequately corrected.^(1)^ Thus, lacking a quantitative measure of motion in real‐time processing, it is not possible to determine whether the motion‐corrected data need to be reacquired. To our knowledge, no such quantitative metric is included in commercial real‐time fMRI packages. The motion problem is compounded in clinical studies, since motion is more common in patients because of paresis, which often leads to recruitment of additional muscles to perform the task, and the presence of artifacts correlates with the degree of paresis.^(1)^

To combat this problem, a number of techniques have been developed to reduce motion artifacts. These include non‐image‐based techniques such as immobilization,^(3)^ ^,^ ^(4)^ although it has been our experience that immobilization of cancer patients can be self‐defeating due to increased patient anxiety. One class of image‐based techniques to reduce motion artifacts involves retrospective image registration and is incorporated into a number of public‐domain postprocessing software packages, including SPM, AIR, and AFNI.^(^ ^5^ ^–^ ^7^ ^)^ Another class involves prospective correction through navigator echoes.^(^ ^8^ ^–^ ^13^ ^)^ A handful of other image‐based techniques have also been discussed in the literature.^(14)^ ^,^ ^(15)^ Because prospective techniques are not readily available on many commercial scanners, the use of image‐registration software packages remains the most widely used approach to motion correction.

Two recent studies compared the image‐registration capabilities of the most widely used packages. Morgan et al. evaluated AIR, AFNI, and SPM on the basis of the number of false positives generated from simulated activation on a synthetic data set.^(16)^ Ardekani et al. compared the motion‐detection algorithms on the basis of the accuracy of motion estimation given fixed amounts of introduced motion.^(17)^ While slight differences were seen in the image‐realignment algorithms, there was no convincing evidence that any of these three registration algorithms were significantly superior.

Because the motion‐correction techniques work best when the motion is small in magnitude, large amounts of motion can render an fMRI experiment useless. It would be highly beneficial for neurosurgical planning applications of fMRI if there were a metric produced in real time that allowed for determination of whether motion in an experiment would be adequately corrected for by standard image‐registration algorithms. Therefore, this study seeks (1) to develop—on a commercial real‐time image‐processing package—a quantitative motion‐detection algorithm to determine the utility of a given data set, and (2) to use the motion‐detection algorithm to evaluate the limits of a standard image‐registration package's performance using a computer‐generated phantom.

## II. MATERIALS AND METHODS

A real‐time motion‐detection module was added to the commercially available Real‐Time Image Processor (RTIP) software (GE Medical Systems, Milwaukee, WI), which was loaded on the NV/*i* Visualization Platform (GE Medical Systems, Milwaukee, WI). The NV/*i* system utilizes a dual processor 296 MHz Sun UltraSPARC 2 computer with 512 MB of memory. The motion detection was performed using a center‐of‐intensity approach. After applying a threshold to eliminate pixels outside the brain, the centers of intensity in the *x*‐, *y*‐, and *z*‐directions are calculated for each time point of an fMRI experiment (typically 14 slices and 65 time points in our implementation). Each center of intensity is calculated in real time. Immediately at the completion of the fMRI experiment, the coefficient of variation (COV) of the center of intensity is calculated and displayed.

To permit assessment of the motion‐detection method with a known amount of introduced motion, a synthetic data set was created from an existing clinical fMRI data set. The functional data set was acquired on a 1.5 T Signa NV/i scanner (General Electric Medical Systems, Milwaukee, WI) with the standard quadrature birdcage head coil. A single‐shot gradient recalled echo planar imaging sequence with a repetition time of 4000 ms and an echo time of 50 ms; a 128×128 matrix size was used to collect the 26 cm field‐of‐view, 6 mm thick images. Fourteen contiguous slices were obtained at 65 time points (five‐time‐point blocks of alternating activation and rest for six cycles), for a total of 910 images. To create the synthetic data set, each slice from the first time point of the data set was duplicated for the remaining 64 time points. Translational, in‐plane rotational (head shaking), and through‐plane rotational (head nodding) motions were introduced to alternate blocks of five time points to mimic stimulus‐correlated motion in a block paradigm study. These synthetic data sets were generated using MatLab software (The Math Works Inc., Natick, MA). Whole‐pixel translations up to nine pixels were introduced in the *x*‐ and *y*‐directions. Rotations with bicubic interpolation were simulated from 0° to 25° in‐plane and from 0° to 6° through‐plane. In‐plane rotations were performed through an axis that runs through the spinal cord.

Each simulated motion data set was evaluated with the motion‐detection algorithm, yielding COV values for the *x*‐, *y‐*, and *z*‐directions. AFNI,^(7)^ a standard offline processing package, was used to correct for motion using a three‐dimensional image‐registration algorithm, *3dvolreg*, which is an iterative method optimized for small translations or rotations.^(18)^ These motion‐corrected data sets were then evaluated by the motion‐detection algorithm to provide a metric for determining the effectiveness of the motion correction. Due to the similar performance of the major image‐registration packages reported by Ardekani et al. and Morgan et al., AFNI alone was used in this analysis.

The replication of time points in the simulated data set implies that there should be no activation found in an fMRI analysis of the data set. However, the presence of motion can create spurious regions of activation, generating a nonzero value for the functional intensity. The intensity of the functional activation is the parameter α in the equation x=αr+a+b+η, where **x** is the data time series vector, **r** is the reference time series vector, *a* is the mean signal, *b* is the linear drift, and η is the error term.^(7)^ The functional intensity is analogous to the percentage signal change. The functional intensity per pixel before and after motion correction was calculated for a central slice to provide a further measure of the limits of acceptable motion correction. One would expect the functional intensity per pixel to reduce to zero for perfect motion correction. This investigation allows direct evaluation of the effect of the motion on the fMRI analysis and how the image‐registration algorithms limit this effect.

AFNI's three‐dimensional image‐registration algorithm was also applied retrospectively to a 20‐patient sample of clinical fMRI runs (56 total data sets). The tasks studied were aurally presented paradigms designed to activate expressive speech receptive speech, and sensorimotor functional centers. The image‐registration algorithm produced the magnitude of the translation and rotation required to realign each slice in the functional data set. This yielded information regarding the amount of motion present in typical aurally presented clinical fMRI runs, and served as a validation of the limits determined in the simulated motion study.

## III. RESULTS

**Figure 1:**
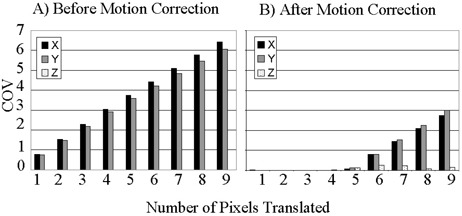
Coefficient of variation (COV) of the center of intensity of the simulated fMRI time course before (a) and after (b) motion correction for translations up to 9 pixels.

**Figure 2:**
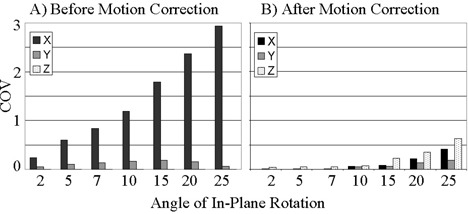
Coefficient of variation (COV) of the center of intensity of the simulated fMRI time course before (a) and after (b) motion correction for in‐plane rotations up to 25°.

**Figure 3:**
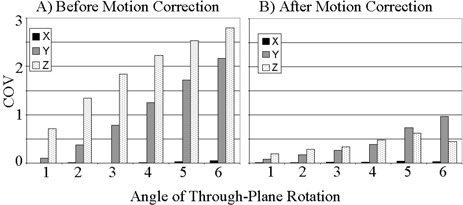
Coefficient of variation (COV) of the center of intensity of the simulated fMRI time course before (a) and after (b) motion correction for through‐plane rotations up to 6°.

The motion‐detection algorithm was successfully implemented within the correlation coefficient module. To measure the speed of the algorithm, it was tested during a real‐time fMRI experiment run on a phantom. The algorithm produced COV values for each of the three planes within 1 s of the end of the experiment. Having verified the ability of the algorithm to process at the necessary speed, the algorithm was applied retrospectively to the simulated motion data sets. Figs. 1 to 3 show the COVs of the *x*‐, *y‐*, and *z‐* centers of intensity obtained for translational, in‐plane rotational, and through‐plane rotational motion both before and after motion correction. The coefficients of variation prior to motion correction increased in a linear fashion, while the postcorrection coefficients of variation were small up to an inflection point. The inflection point occurred for a translation of 5 pixels in Fig. 1 and an in‐plane rotation of 10° in Fig. 2, but was less evident in the through‐plane rotation depicted in Fig. 3. Fig. 2(a) shows a greater COV along the *x*‐axis than along the *y*‐axis. This is likely due to the fact that for small angles of in‐plane rotation of an oblong object such as the brain, the dominant shift in the center of intensity is likely to be perpendicular to the long axis of the object. Likewise, Fig. 3(a) shows a larger COV along the *y*‐axis because a through‐plane rotation is likely to show very little shift along the *x*‐axis since the axis of rotation is along the same axis.

**Figure 4:**
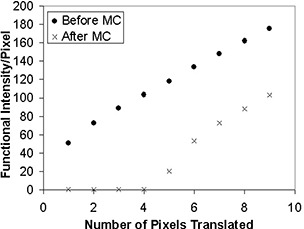
Functional intensity of the simulated fMRI time course per pixel before and after motion correction (MC) for translations up to 9 pixels.

**Figure 5:**
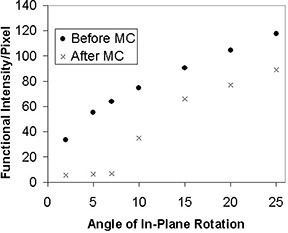
Functional intensity of the simulated fMRI time course per pixel before and after motion correction (MC) for in‐plane rotations up to 25°.

**Figure 6:**
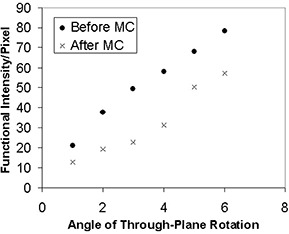
Functional intensity of the simulated fMRI time course per pixel before and after motion correction (MC) for through‐plane rotations up to 6°.

Figs. 4 to 6 show the functional intensity per pixel for translation, in‐plane rotation, and through‐plane rotation before and after motion correction. It is readily apparent that the simulated stimulus‐correlated motion generates an appreciable amount of spurious activation. However, the motion‐correction algorithm removes this spurious activation for small magnitude translations and rotations, as evidenced by the low postcorrection functional intensity values in this domain. An inflection point was again present in each type of motion, implying that at motion levels beyond that inflection point there was a marked decrease in the ability of the software to remove the spurious activation. Inflection points occurred at approximately 5 pixels for translation, 10° for in‐plane rotation, and 5° for through‐plane rotation. The data reflect the fact that AFNI's image‐registration algorithm is designed for translations of only a few pixels and for rotations of only a few degrees.^(18)^

The inflection point values for functional intensity were consistent with the values from the COV analysis. Following analysis of the motion‐detection parameters at these points, it was determined that the quadrature sum of the *x*‐, *y*‐, and *z*‐coefficient of variation values is a good metric for identifying the inflection points for all three types of motion. Based on the results of the synthetic data analysis, a COV quadrature sum of 2.0 pixels corresponds to the threshold of correctability for the three types of introduced motion. Thus, it is recommended that any fMRI data set showing a COV quadrature sum greater than 2.0 pixels be reacquired to obtain a data set with less motion corruption.

The analysis of motion in the 20 clinical data sets revealed that the maximum rotation required to align a slice was 1.4°, while the maximum translation required was 2.3 mm (about 1 pixel). The fact that the maximum rotation and translation were well below the inflection points within which motion correction was possible indicated that these clinical data sets would be expected to contain correctable amounts of motion.

## IV. DISCUSSION

The real‐time motion‐detection algorithm was selected and implemented in a manner that sought to minimize the calculation time during the run. More intricate motion‐detection approaches were considered, but they would have required greater allocation of memory and processor time. The center‐of‐intensity method was efficient enough to operate in the real‐time processing environment and generated results within one second of the end of the fMRI run. Thus, the degree of patient motion can be evaluated allowing an immediate decision regarding whether to reacquire the data. This is particularly useful due to the increased likelihood of motion in patients relative to healthy volunteers, and the impracticality of recalling surgical patients in a busy clinical setting where surgery may be performed within 24 h of the fMRI procedure.

Synthetic data sets were employed during the validation of the algorithm because they were simple to create and allowed for the introduction of known amounts of motion of any type at any point in the time series. We chose to emphasize stimulus‐correlated motion because this is the type of motion most troublesome to fMRI data analysis and is common in clinical fMRI.

It is worth noting that in Figs. 1 and 2, which show results after in‐plane motion was introduced, a *z*‐component for the COV appeared after motion correction. This is explained by the fact that the motion‐correction algorithm uses scaling and rotations in all directions to align the images. This may introduce variations of the center of intensity in the *z*‐direction. An additional observation of the phantom analysis is that the degree of correction of through‐plane motion was far less than the degree of correction for in‐plane rotation or translation. This is likely due to the fact that the *z*‐axis dimension of 6 mm is far greater than the approximately 2 mm in‐plane voxel dimension. Thus, interpolation errors contribute preferentially to through‐plane motion correction.

The motion analysis of the 20 previously acquired fMRI cases provided an estimate of the amount of motion seen in typical clinical fMRI data sets. No motion that exceeded our threshold of correctability was present in these data sets. However, the paradigms analyzed were all aurally presented. Motion may be more likely in visually presented paradigms, in which the patient may adjust his or her head to better view the stimulus. Additionally, the data sets were biased toward being relatively free of motion artifacts, because they were selected from a database containing data sets presented to the neurosurgeons for presurgical planning. Other fMRI applications may have a higher incidence of rejected data sets than the ones investigated here.

In conclusion, we have implemented a real‐time motion‐detection algorithm and have established the motion‐detection values that define the threshold of correctability, allowing a decision to be made within one second of the end of the experiment regarding whether data reacquisition is necessary. Clinical implementation of the procedures used in these studies would be expected to reduce the need to recall patients at a later time and limit the number of fMRI runs discarded because of uncorrectable motion artifacts. The overall result of this work is the addition of a tool to augment the ability of real‐time fMRI data processing to assist in neurosurgical planning.

## ACKNOWLEDGMENTS

This work was funded in part by the John S. Dunn Foundation. The support of William A. Murphy, Jr., MD, is gratefully acknowledged.
